# Is a Hybrid Pulmonary Rehabilitation Programme Feasible and Effective in Individuals With COPD After an Exacerbation‐Related Hospitalisation: A Mixed Methods Study

**DOI:** 10.1111/jocn.70234

**Published:** 2026-02-04

**Authors:** Sarah Gephine, Elise Meto, Olivier Le Rouzic, Robert Launois, Jean‐Marie Grosbois

**Affiliations:** ^1^ FormAction Santé Pérenchies France; ^2^ Univ. Lille, Univ. Artois, Univ. Littoral Côte d’Opale ULR 7369 ‐ URePSSS ‐ Unité deRecherche Pluridisciplinaire Sport Santé Société Lille France; ^3^ Réseau d'Evaluation en Economie de la Santé Paris France; ^4^ Univ. Lille, CHU Lille, CNRS, Inserm, Institut Pasteur de Lille U1019—UMR 9017—CIIL—Center for Infection and Immunity of Lille Lille France

**Keywords:** chronic obstructive pulmonary disease, exacerbation, home, hospitalisation, perspective, qualitative, telerehabilitation

## Abstract

**Aim:**

To combine qualitative and quantitative data to evaluate the feasibility, participant satisfaction and effectiveness of a hybrid pulmonary rehabilitation programme following hospital discharge for an exacerbation of chronic obstructive pulmonary disease (COPD).

**Design:**

Convergent parallel mixed method study nested in a larger ongoing prospective study; this report includes a subset of 21 participants who complete the qualitative and quantitative assessments between May 2023 and January 2024.

**Methods:**

Semi structured interviews using open‐ended questions were conducted and analysed using a thematic analysis approach. Participants were interviewed after completing an 8‐week hybrid home‐based rehabilitation programme, including four face‐to‐face and four remote sessions. Quantitative assessments—covering disease impact, anxiety and depressive symptoms, and exercise tolerance—were conducted at the beginning and end of the intervention in the same participants who took part in the interviews, and a 10‐item satisfaction questionnaire was also completed after the programme.

**Data Sources:**

May 2023 to January 2024.

**Reporting Method:**

GRAMMS checklist was followed.

**Results:**

21 people with chronic obstructive pulmonary disease (11 females; mean age 62 ± 7 years; mean FEV_1_ 30% ± 10% of predicted) were interviewed. Five major themes were identified: (i) accessibility and adaptation to individual needs; (ii) confidence in the transdisciplinary care manager model, confirmed by high satisfaction score (95/100); (iii) integration of informal carers; (iv) perceived benefits supporting maintenance of health behaviour, consistent with the statistically and clinically significant improvements observed across all quantitative outcomes; and (v) hybrid programme challenges (technical issues and preference for face‐to‐face visits).

**Conclusion:**

The hybrid programme resulted in significant improvements in physical and psychological outcomes, and participants reported high levels of satisfaction. Qualitative findings highlighted the value of home‐based delivery, supervision by a single care manager, informal carer involvement and emotional support in shaping feasibility and satisfaction. However, challenges related to remote sessions indicate that telerehabilitation may not be suitable for all patients and should not be used as a standalone PR option.

**Relevance for Clinical Practice:**

Given the strong preference of participants for face‐to‐face visits over remote visits, telerehabilitation should always include a minimum of individual or group face‐to‐face supervised sessions. The balance between supervision modalities should be personalised according to participants' needs and progress.

## What Does This Paper Contribute to the Wider Global Clinical Community?

– Exploring patients' experiences with hybrid home‐based pulmonary rehabilitation provides valuable insights to guide the design and implementation of future programmes.
– Patients emphasised the importance of having a single transdisciplinary healthcare referent (i.e., a care manager), which facilitated a trusting and supportive therapeutic relationship.
– Participants reported technical challenges during telerehabilitation sessions and unanimously expressed a preference for face‐to‐face, supervised sessions over remote options.

## 1 Introduction

People with chronic obstructive pulmonary disease (COPD) are at high risk of severe acute exacerbations (AECOPD), defined as acute events characterised by a worsening of respiratory symptoms that require changes to the patient's usual treatment (MacLeod et al. 2021). AECOPD leading to hospitalisation represents a substantial social, psychological and economic burden for patients, informal carers and healthcare systems (Halpin et al. 2017), and increases the risk of rehospitalisation and mortality during the year following discharge (Waeijen‐Smit et al. 2024). In addition, a severe limitation in daily‐life activities, potentially leading to feelings of anger, psychological distress and reliance on family members, is reported (Machado et al. 2022). Therefore, it is essential to provide personalised support to these individuals upon their discharge from hospital.

## 2 Background

Pulmonary rehabilitation (PR) is recognised as a highly effective treatment for people with stable moderate to severe COPD, regardless of the delivery modality (Cox et al. 2021; McCarthy et al. 2015; Vieira et al. 2010; Wadell et al. 2013). PR is defined as an integrated person‐centred intervention, including education, self‐management strategies and exercise training, to promote long‐term adherence to health‐enhancing behaviours (Spruit et al. 2013). Recent studies report that PR is also effective in improving quality of life and exercise capacity, and for reducing hospital readmissions and dyspnea in people discharged from a hospitalisation for AECOPD (Jenkins et al. 2024; Puhan et al. 2016). It is now strongly recommended to initiate a PR programme within 3 to 4 weeks posthospital discharge (Jenkins et al. 2024; Rochester et al. 2023). Nevertheless, the referral and completion rates remain extremely low: 2.7% in the USA and 9.6% in England 12 months after discharge (Jones et al. 2014; Spitzer et al. 2019), and 8.6% in France within 90 days after discharge (Guecamburu et al. 2023). Barriers that negatively affect PR uptake, included the number of beds in out or inpatient PR, the number of ambulatory places, and the distance to PR centres (> 16 km) (Guecamburu et al. 2023; Spitzer et al. 2019). While PR programmes are commonly outpatient‐based and delivered on a twice‐ or thrice‐ once‐weekly basis during 4 to 8 weeks (Spruit et al. 2014), home‐based and telerehabilitation programmes have the potential to enhance PR uptake with similar benefits (Cox et al. 2021; Maltais et al. 2008; de Mens Oliveira et al. 2010; Vieira et al. 2010). Telerehabilitation involves the use of information and communication technologies to deliver rehabilitation services remotely and must include all essential components to be considered an effective alternative to conventional PR (Cox et al. 2021). When supervised sessions are provided in telerehabilitation programmes, they are typically conducted via video or phone calls. It is important to distinguish between remotely supervised sessions using telecommunications tools and face‐to‐face visits conducted at the patient's home. Hybrid interventions combine supervised face‐to‐face visits (whether at the PR centre or at the patient's home) with synchronous or asynchronous telerehabilitation sessions.

As ‘one size does not fit all’ (Holland et al. 2021), we have developed over the last 15 years a personalised home‐based PR programme for people with chronic respiratory disease that includes one weekly face‐to‐face supervised session at the patient's home during 8 weeks (Grosbois et al. 2015). This home‐based programme is characterised by three key innovative aspects that, to the best of our knowledge, are unique among existing PR settings: (1) patient care is delivered at home by a single healthcare professional (i.e., a care manager); (2) the PR team functions within a transdisciplinary framework; and (3) all assessments are conducted at the patient's home.

By definition, PR teams involve health professionals from diverse multidisciplinary backgrounds, most commonly physiotherapists, nurses, dietitians, kinesiologists and respiratory or chest physicians (Spruit et al. 2014). In practice, most PR teams have adopted a multidisciplinary model—where each discipline focuses solely on its specific role—or, at best, an interdisciplinary/interprofessional model, in which professionals exchange information but remain primarily responsible for care delivery within their own discipline (Campagna and Nelson 2019; Reilly 2001; Wikman et al. 2025). These siloed approaches, characterised by parallel roles and limited shared responsibility for patient‐centred goals, have been shown to be less effective (Campagna and Nelson 2019; Gordon et al. 2014; Reilly 2001). Transdisciplinary models, in contrast, promote shared understanding, overlapping competencies and collective responsibility for patient goals, with professional boundaries intentionally crossed to deliver more integrated and person‐centred care (Campagna and Nelson 2019; Gordon et al. 2014). In such models, healthcare professionals educate one another across disciplines, enabling flexible care delivery that adapts to individual needs rather than discipline‐specific tasks. It actively promotes and empowers patients and their carers to collaborate with healthcare professionals in determining and negotiating an individualised action plan aimed at achieving their personal goals.

To address the complexity of managing individuals with chronic respiratory disease and the logistical challenges of delivering PR at home across a large and diverse region of northern France, we developed the care manager concept as a core component of our home‐based PR programme. In this model, a single healthcare professional delivers the full intervention—including education, psychosocial support and exercise training—regardless of their initial professional background. To enable this, care managers receive ongoing standardised training and participate in weekly peer exchanges that promote shared competencies across disciplines (Gephine et al. 2026).

While many home‐based rehabilitation programmes are now conducted at patients' homes via telecommunication tools (telerehabilitation), pre‐ and post‐assessments are typically performed at the referring hospital (Cox et al. 2021). In our model, assessments are performed at the patient's home to include individuals who are geographically or socially isolated, who work or who find travel difficult. For this reason, we use the 6‐min stepper test—a validated measure of exercise capacity in patients with COPD (Grosbois et al. 2016)—as the 6‐min walk test requires a corridor length not typically available in homes.

During the COVID‐19 pandemic, our usual home‐based PR programme was delivered entirely remotely, similar to outpatient centres (Grosbois et al. 2021). Building on this experience, we developed a hybrid programme combining face‐to‐face and synchronous telerehabilitation sessions for patients with COPD recently discharged for an exacerbation. This mixed‐methods study (May 2023–January 2024) was embedded within a larger prospective study of the hybrid programme, initiated in January 2022, with the complete quantitative dataset expected by March 2026. While essential, quantitative analysis alone is insufficient to ensure sustained and widespread implementation. To support broader adoption, it is necessary to understand the factors contributing to success or failure through feedback from patients and professionals. Identifying these facilitators and barriers is crucial for adapting the programme to diverse contexts and meeting patients' needs. Incorporating patient perspectives is essential to inform programme design but also to engage policymakers and secure funding for its implementation.

## 3 Aims

This convergent parallel mixed‐methods study aimed to combine qualitative and quantitative data to assess the feasibility, participant satisfaction and effectiveness of a hybrid pulmonary rehabilitation programme following hospital discharge for COPD exacerbation.

## 4 Methods

### 4.1 Study Design and Participants

This convergent parallel mixed‐methods study was embedded within a larger ongoing prospective study evaluating the effectiveness of a hybrid PR programme. The complete dataset is expected to be available by March 2026. To date, no original research articles have been published using this dataset. The prospective study initiated in January 2022, while this mixed‐methods study was conducted between May 2023 and January 2024. Although the initial protocol was primarily designed to explore qualitative data, quantitative data were also collected to characterise the sample, contextualise the findings and support the perceived benefits reported by participants. To facilitate the broader implementation of hybrid PR programmes within the French healthcare system, mixed‐methods studies are essential for capturing the comprehensive effectiveness of the intervention across both experiential and quantitative dimensions.

Human Research Ethics approval was provided and all participants provided written informed consent for the utilisation of their data for research purposes.

Individuals who experienced a severe AECOPD were referred to the 8‐week hybrid PR programme by their respiratory physicians. The physicians were responsible for confirming the diagnosis of COPD according to the Global Initiative for Chronic Obstructive Lung Disease (GOLD) classification system and ensuring the absence of cardiovascular contraindications to exercise training. They also presented and offered the available PR modalities to each participant. Eligibility required a diagnosis of severe AECOPD and completion of PR. Exclusion criteria included any cognitive or mental disorders that could have influenced the interviews. Sociodemographic (age, sex), anthropometric (body mass index [BMI]) and general clinical data (lung function, use of long‐term oxygen therapy [LTOT] and non‐invasive ventilation [NIV]) were collected from the individual's medical record to characterise the sample.

### 4.2 Description of the Intervention

**FIGURE 1:**
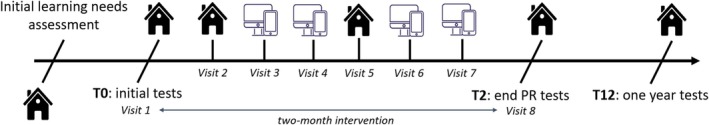
Design of the hybrid pulmonary rehabilitation programme. [Colour figure can be viewed at wileyonlinelibrary.com]

All participants performed an 8‐week personalised home‐based PR programme, comprising a weekly supervised 90‐min supervised session. The hybrid programme comprised four face‐to‐face sessions conducted at the patient's home by the care manager (sessions 1, 2, 5 and 8) and four remotely supervised synchronous telerehabilitation sessions delivered via videoconference or telephone, depending on the participant's digital accessibility (sessions 3, 4, 6 and 7) (Figure 1).

A learning needs and goals assessment was conducted at the patient's residence before the beginning of PR. This assessment informed the design of a personalised intervention, created through a collaborative process between the care manager, the participant and their informal carer (if present). In addition to the weekly supervised home visit, participants were required to engage in personalised physical training (a minimum of four sessions per week) and self‐management plan for the remainder of the week.

Education and self‐management interventions were adapted to respond to the specific needs, barriers and personal goals of each individual. The core educational topics included the prevention and recognition of exacerbations as participants were referred after an exacerbation related‐hospitalisation; pathophysiology of lung disease and comorbidities, medication and its use, indoor air pollution, physical activity, breathing techniques, stress management and emotional responses related to the disease. Other topics could be addressed according to the participant's needs including nutritional counselling, smoking cessation strategies, help with ventilatory support setup, airways clearance techniques, relaxation techniques and end‐of‐life planning.

Each participant was provided with a cycle ergometer (Domyos 120, Decathlon, Villeneuve‐d'Ascq, France) and/or a stepper (Go Sport, Grenoble, France), and/or a mini bike (Domyos 100, Decathlon, Villeneuve‐d'Ascq, France). The selection and implementation of the equipment were negotiated between the care manager and the patient, considering the patient's abilities, needs, preferences and home environment accessibility. For endurance training, the goal was to achieve a total of 30–45 min of daily exercise, performed in 10‐min intervals or shorter depending on individual capacity, with at least five sessions per week—one supervised (either face‐to‐face or remotely synchronous) and four performed independently by the patient. Exercise intensity was progressively adjusted to reach a dyspnea score between 3 and 4 (moderate to somewhat severe) on the Borg 0–10 scale or 11–13 on the Borg 6–20 scale (Borg 1982). Physical training was completed with upper and lower limb muscle strengthening exercises using dumbbells, elastic bands, swissball and/or body weight on the same daily basis as cardiorespiratory training. Intensity was gradually adjusted (increasing the number of repetitions and/or resistance) according to participant's experience of dyspnœa or fatigue. Oxygen saturation and heart rate were monitored using a handheld pulse oximeter. For the participants with the most severe deconditioning who were unable to tolerate cardiorespiratory training, the intervention started with two 30‐min daily sessions of self‐administered quadriceps electrostimulation, five times a week (Vivodtzev et al. 2006). All participants were encouraged to increase the amount of time spent in daily life physical activities such as gardening, housekeeping, groceries, in order to facilitate the integration of long‐term physical activity (Gephine et al. 2021). Physical activity and/or exercise training maintenance strategies were discussed and negotiated between the patient and the care manager (and the informal carer if applicable) throughout the course of the 8‐week programme.

### 4.3 Description of the Care Manager Concept

Each participant received the complete PR programme from a single care manager, regardless of the care manager's initial professional background (Gephine et al. 2026). After receiving a prescription from the respiratory physician, participants were assigned to a care manager based on geographical proximity, ensuring coverage across urban and rural areas of northern France. The PR team comprised a respiratory physician (medical coordinator), two physiotherapists, three nurses, four kinesiologists, a psychologist, a dietician, an art therapist and a sociomedical beautician. Despite diverse initial expertise, all team members completed the same standardised 40‐h therapeutic education training and training in behaviour change and motivational communication techniques. New care managers underwent a two‐month mentorship period, working alongside senior care managers in patients' homes to acquire skills beyond their original training.

To function as a transdisciplinary team, weekly four‐hour meetings were held, where care managers presented new patients and proposed action plans. Specialists provided advice across disciplines as needed, and sessions included ongoing training by team members or external experts (e.g., palliative care). This approach enabled care managers to deliver all aspects of the PR programme at patients' homes. When necessary, care managers could consult colleagues with specific expertise; for example, nurses could request kinesiologists' advices about a specific training exercise or physiotherapists could request respiratory physician input for persistent exacerbations. The assigned care manager was the sole point of contact for the patient and remained accessible by phone outside weekly visits.

### 4.4 Interviews

Individual semi‐structured interviews with open‐ended questions were conducted by two independent specialists uninvolved in the intervention's design or delivery. After the hybrid PR programme, the PR team compiled a list of 35 participants who completed all 8 sessions and consented to interviews. From this list, 28 participants were randomly selected and contacted.

Based on deductive thematic analysis, interviews lasted 30–45 min and explored possible facilitators and barriers to adherence, personal perspectives on the intervention's design, and questions about benefits and long‐term maintenance (Table 1).

**TABLE 1:** Interview questions.

**1. Prescription, choice, expectations**
How did you hear about the home‐based PR programme? What made you think your physician would refer you to this intervention? Have you experienced any challenges or barriers for enrolling in the hybrid programme? What were your initial expectations regarding this intervention?
**2. Design and implementation of the hybrid intervention**
Was the rehabilitation programme designed with you? Was it adapted to meet your needs? What did your PR programme include? Were the exercises diversified and progressively adapted? Were you able to get all the support you needed from the care manager during the sessions? Was there a difference between the face‐to‐face sessions and the telerehabilitation sessions? Did you prefer one over the other? Were you able to complete the remote sessions successfully? Did your carer attend the PR sessions? If so, did she/he benefit from it?
**3. Benefits, maintenance and satisfaction**
Have you noticed any improvement in your physical and/or mental health after the programme? At the end of PR, was a personalised programme provided to help you maintain positive behaviours? Have these behaviours been maintained? Was the number of rehabilitation sessions provided enough? What is your overall satisfaction with the hybrid home‐based programme?

### 4.5 Data Analysis

All interviews were audio taped and transcribed verbatim, with transcripts checked for accuracy by an author (E.M.). All transcripts were de‐identified. Qualitative content analysis was carried out to generate codes and assign categories using ATLAS Ti 9.0 software (Scientific Software Development GmbH, Berlin, Germany) by two authors (S.G. and E.M.). As preconceived themes were expected to be found, a deductive thematic analysis was used while still maintaining flexibility to allow for the generation of new themes. Two authors (S.G. and J.M.G.) reviewed the themes and coding, and an agreement on major themes and subtheme was found with a third author (E.M.). Data collection continued until saturation was achieved. Representative quotes were included to support the interpretation of the identified themes and subthemes. For the purpose of publication, quotes were translated into English from French.

### 4.6 Validity, Trustworthiness, Rigour and Reflexivity

To ensure the validity and trustworthiness of the qualitative analysis (Malterud 2001), participants were randomly selected from those who completed the programme to minimise selection bias. Data collection continued until thematic saturation was reached, ensuring comprehensive coverage of relevant perspectives. Researcher triangulation was applied, with two researchers independently analysing each interview and developing codes, which were then compared and refined collaboratively with a third researcher to ensure accurate representation and resolve discrepancies. To enhance transparency, representative participant quotes were included to illustrate and support the interpretation of identified themes and subthemes. Rigour was maintained by employing a primarily deductive thematic analysis approach, complemented by qualitative content analysis to generate codes and assign categories. The analysis was conducted using a specific software (ATLAS.ti 9.0) by two authors. While preconceived themes guided the deductive approach, flexibility was preserved to allow for the emergence of novel themes, ensuring the analysis remained grounded in the data. To mitigate potential researcher bias, interviews were conducted by external interviewers not involved in the intervention delivery. Regular team discussions were held to reflect on researcher positioning, assumptions and the interpretation of data. Methodological decisions, including the choice of a primarily deductive thematic analysis, were continuously reviewed and aligned with the research questions and contextual factors.

### 4.7 Quantitative Assessments

To support the qualitative analysis, a quantitative assessment of the impact of the disease on the patient's life, exercise capacity and anxiety and depressive symptoms was performed with the same participants who took part in the interviews. All the assessments were performed at the participants' home at the beginning and at the end of PR.

The impact of COPD was assessed using the *COPD Assessment Test* (*CAT*) a validated tool specifically developed for patients with COPD (Jones et al. 2009). The validated French version of the CAT was used in this study (Marchand and Maury 2012). This self‐administered questionnaire consists of eight items covering various symptoms and consequences of COPD, providing a simple and quantifiable measure of health‐related quality of life. Each item is scored on a scale from 0 to 5, resulting in a total score ranging from 0 to 40, with higher scores indicating a greater impact of the disease on the patient's life. The CAT has demonstrated excellent internal consistency in previous studies, with a Cronbach's alpha ranging from 0.85 to 0.98 (Gupta et al. 2014). CAT is also sensitive to changes following PR and a minimal clinically important difference (MCID) of −2 points has been reported (Kon et al. 2014).

Anxiety and depressive symptoms were assessed using the self‐administered Hospital Anxiety and Depression Scale (HADS), which consists of 14 items rated on a 4‐point Likert scale (0–3) (Zigmond and Snaith 1983). The validated French version of the HAD was used in this study (Lepine et al. 1985). The scale is divided into two subscales, each containing seven items: one for anxiety and one for depression. The total score ranges from 0 to 42, while each subscale score ranges from 0 to 21. Higher scores indicate a greater likelihood of anxiety or depressive symptoms. A subscale score < 7 indicates no symptoms, 8–10 suggests possible anxiety or depression, and > 11 indicates a probable clinical diagnosis requiring further evaluation. The HAD has demonstrated good to excellent internal consistency in previous studies, with Cronbach's alpha values ranging from 0.73 to 0.87 (Nikolovski et al. 2022). It is also responsive to changes following PR, with a reported MCID of −1.5 points for each subscale (Puhan et al. 2008).

Exercise tolerance was assessed using the 6‐min stepper test (6MST), a validated tool for patients with COPD (Grosbois et al. 2016). As all assessments were conducted in participants' homes, the 6MST was chosen over the 6‐min walk test, which requires a 30‐m corridor. Before the test, all participants underwent a 2‐min familiarisation session with the stepper. Standardised instructions were provided, instructing participants to complete the maximum number of steps possible within 6 min. A ‘step’ was defined as a full movement of lifting and lowering 1 ft. No verbal encouragement was given during the test. For participants with balance issues, light manual support against a wall or piece of furniture was permitted. The 6MST is responsive to changes following pulmonary rehabilitation, with a reported MCID of +40 steps (Pichon et al. 2016).

Participants also complete a 10‐item satisfaction questionnaire at the end of the hybrid programme. Each item was scored on a 0–10 Likert scale (0 = very poor and 10 = excellent), giving a total score ranging from 0 to 100 (higher indicates greater satisfaction). Two questions addressed satisfaction with the duration and frequency of the programme sessions, four questions focused on the care manager concept, two questions concerned the programme itself and two questions addressed perceived benefits.

Quantitative data were analysed using IBM SPSS Statistics V30.0 (IBM Corp., NY, USA). To evaluate changes in quantitative outcomes over time, paired‐*t*‐tests were performed, as data were normally distributed. Results are presented as mean change (SD), and the level of significance was set at 0.05.

## 5 Results

**FIGURE 2:**
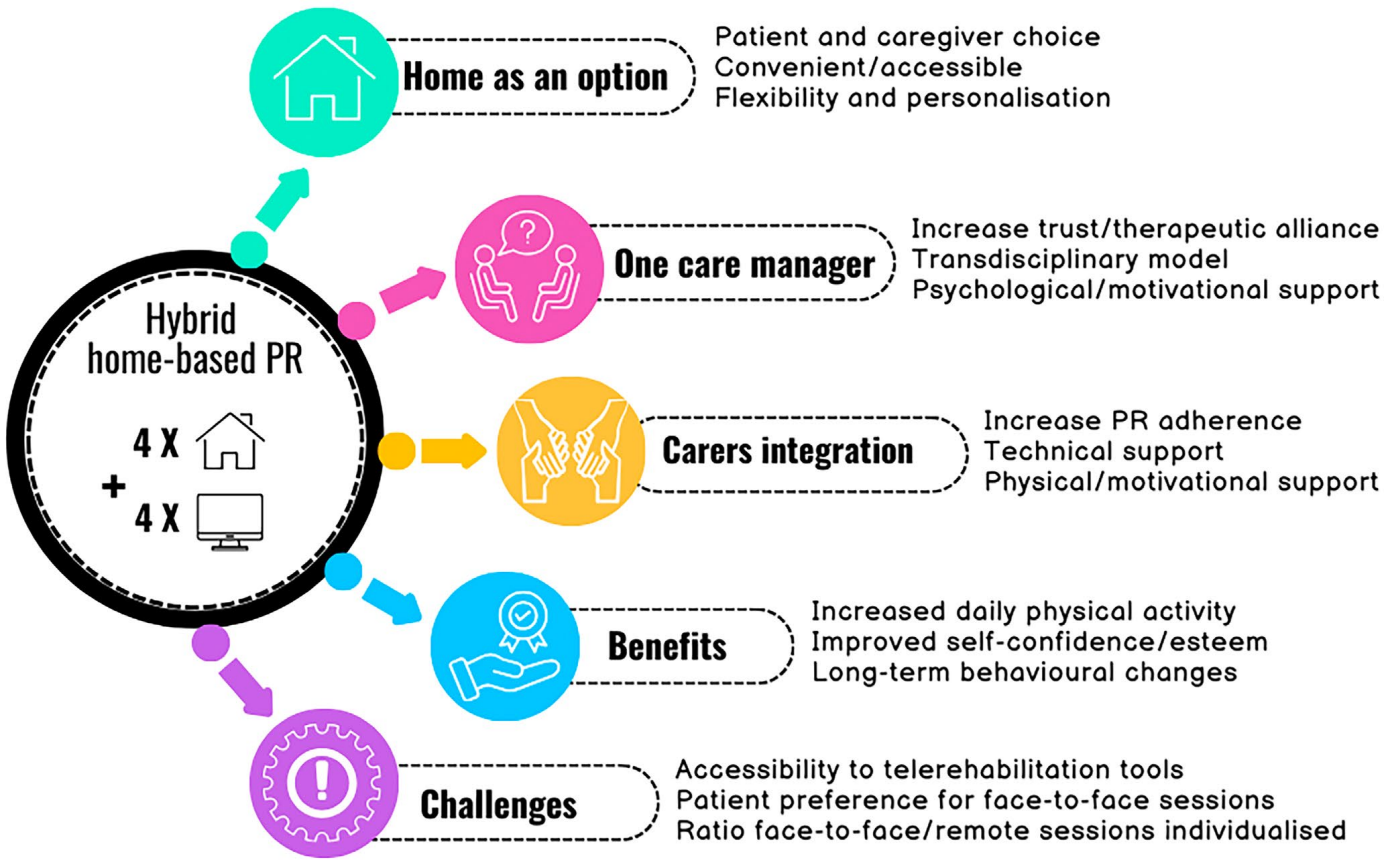
Diagram summary of patient experiences following completion of the hybrid home‐based pulmonary rehabilitation programme. [Colour figure can be viewed at wileyonlinelibrary.com]

Between May 2023 and January 2024, a total of 28 individuals were invited to participate, of whom 21 consented to be interviewed (Table 2). All the interviews were conducted through telephone. Most participants were married (17, 80.9%), 17 (80.9%) participants had a carer living with them, 12 (57.1%) required LTOT and 8 (38.1%) required NIV. At the beginning of PR, 6 participants were too frail to perform the 6MST. On average, the participants were enrolled 6 (5) weeks after being discharged from hospital. Table 3 summarises the four major themes identified by participants as contributing to the success of the hybrid PR programme. A fifth theme covers the challenges faced by participants during its implementation. The main findings related to these five themes are presented in Figure 2. Overall satisfaction was excellent with a mean score of 95/100.

**TABLE 2:** Sociodemographic, anthropometric and general clinical data of the participants.

Patient	Age (years)	Sex	BMI (kg/m^2^)	FEV_1_, % predicted	Baseline CAT	Post PR CAT	Baseline 6MST, steps	Post PR 6MST, steps	Baseline HADS	Post PR HADS	Weeks between discharge and PR
P01	62	Female	21.4	22	24	16	314	396	19	19	14
P02	76	Male	22.8	21	24	17	NA	NA	24	15	11
P03	67	Female	15.4	20	27	16	NA	NA	27	19	2
P04	55	Male	26.6	27	10	9	634	996	15	9	3
P05	64	Male	26.9	34	21	16	226	268	17	12	3
P06	49	Male	29.1	27	20	17	652	702	22	15	17
P07	47	Male	24.1	18	19	17	NA	494	19	14	2
P08	70	Female	37	40	33	29	288	380	20	13	12
P09	53	Female	35.9	19	27	24	146	140	22	4	1
P10	62	Female	22.4	36	32	25	280	386	20	19	2
P11	61	Female	16.2	50	29	18	388	494	20	11	4
P12	57	Female	16.7	28	18	10	244	302	16	18	10
P13	46	Female	22.7	15	30	24	128	148	35	33	23
P14	57	Male	23.6	29	19	20	346	368	9	8	1
P15	65	Female	16.7	32	34	38	NA	NA	28	32	2
P16	68	Male	18.9	15	24	19	NA	NA	16	9	4
P17	67	Male	16.5	33	25	18	206	190	24	19	3
P18	73	Male	38.5	52	33	22	474	628	24	16	5
P19	69	Female	28.2	37	12	6	260	380	6	3	4
P20	71	Male	25	59	15	9	440	628	17	7	7
P21	62	Female	20.3	20	25	12	NA	NA	30	10	2
Mean (SD)	62 (8.5)	11F/10M	24.0 (6.8)	30.2 (12.3)	23.9 (6.9)	18.2 (7.4)	335 (158)	431 (225)	20.5 (6.6)	14.5 (7.7)	6.3 (6.0)

Abbreviations: 6MST, 6‐min stepper test; BMI, body mass index; FEV1%, forced expiratory volume in 1 s as percentage predicted; NA, participant could not perform the test.

**TABLE 3:** Major themes derived from the interviews.

1. Accessibility and adaptation to individual needs
2. Confidence in the transdisciplinary care manager model
3. Integration of the informal carers
4. Benefits encouraged maintenance in behaviour changes
5. Hybrid PR programme challenges

### 5.1 Theme 1: Accessibility and Adaptation to Individual Needs

The flexibility and accessibility of the intervention strongly emerged as a factor of success. A strong preference for the home programme over the centre one was indicated by 8 participants when the choice was offered by their respiratory physicians. The participants cited the well‐known barriers associated with centre PR as factors that influenced their selection for the home programme (Table 4). Only two participants who previously enrolled in an outpatient programme indicated that they would have preferred this option again. The main reasons given were the perception of insufficient supervision and the paucity of training equipment at home.

**TABLE 4:** Factors influencing participants' choice for the home programme.

**To avoid another hospitalisation** I did not want to be hospitalised again (inpatient PR), so doing it at home was a great option for me. It gave me the support I needed when I left the hospital. (P3) I was really pleased to hear about the option at home because I find it difficult to leave my house these days. Since my last hospitalisation, I have developed social anxiety. (P13)
**To avoid transportation** The best thing about it is that you don't have to leave home to get the care you need. (P14)
**To maintain family and professional life** You're not in a center away from home and your family for 4 or 5 weeks. (P14) My job was important to me, and I didn't want to take time off to be hospitalised. (P12)
**To be supported after the hospitalisation as a result of their frail status** I wasn't well enough to be on my own at home. I was still weak and struggling to breathe, so the support I got from the home programme was really helpful and reassuring. (P3) I was very underweight, with no muscle mass left. (P11) I also been diagnosed with prostate cancer and cervical problems […], and I was stressed with all these health issues. (P17)

Despite the participants' multiple comorbidities and frailty status, there was consensus that the intervention met their needs. Personalised care and adapted physical exercises facilitated their engagement in the intervention despite their physical limitations.

A personalised program was designed for me, and I feel it was particularly adapted to my situation, especially given my lack of exercise up to now. (P1)

In addition, participants expressed satisfaction with the integration of their personal expectations into the programme.

I have been involved in the design of my rehabilitation programme and, thus, it met my needs. (P12)

### 5.2 Theme 2: Confidence in the Transdisciplinary Care Manager Model

Three factors were identified as contributing to satisfaction and success (Table 5). The involvement of a single healthcare professional (i.e., a care manager) built trust and a strong therapeutic alliance. Due to the transdisciplinary approach, 80% of participants indicated that they were unaware of the professional title of their care manager, without affecting the quality of the care provided. None of the participants expressed a wish to receive the PR programme from different healthcare professionals. In addition to the exercise training support, 17 (81%) participants emphasised the importance of psychological support as part of the PR programme. Participants felt fully listened to and supported, boosting their commitment and motivation throughout the intervention. This was supported by quantitative data from the satisfaction questionnaire, with a mean score of 10/10 for each item related to the care manager concept.

**TABLE 5:** Factors promoting a successful therapeutic alliance and the effectiveness of the intervention.

**A single care manager** I'd rather stick with the same person because we had done a full interview, including medical and psychological needs. I didn't want to have to keep going over my conditions to someone new every time. (P11) I think it's important to have one person to talk to for the whole process. If the connection wasn't good, I might have stopped. (P15)
**A transdisciplinary model** I don't know what her specialty was. She could have been a physiotherapist, nurse or doctor. (P5) I don't know her profession, but she was well‐trained. (P1) I received a full rehabilitation with breathing exercises, instruction on how properly use my medications, and I also learned how to manage my respiratory equipment. (P10)
**A psychological and emotional support** We had open conversations and I really needed that emotional support. (P11) She arrived when I was going through a tough time. She helped me a lot by being kind, understanding, and encouraging me. (P21) Her job title wasn't important; what mattered was how well she listened, how she supported me, and how sincere she was. (P8)

### 5.3 Theme 3: Integration of the Informal Carers

Ten (47.6%) participants reported that their carer was actively involved during the hybrid programme, leading to a positive impact on participant programme adherence. The presence of the carer was perceived as a source of emotional and physical support, helping the participant complete exercise training apart from supervised visits.

My husband was always by my side. He did the exercises with me, to support me. (P8)

When my coach (care manager) was not here, I was doing the exercise with my wife. It made me feel safer and more motivated.

Carers could also provide significant assistance in the setup of the videoconference sessions.

My wife has a smartphone, and she set up the video sessions. (P15)

Integrating the carer into the intervention also had beneficial effects on their own physical and psychological health.

Yes, my wife is still involved; it's a good idea—it keeps her in shape as well. (P5)

I was able to participate in activities and get psychological support; she helped me emotionally, and I wasn't left out. I really appreciated that. (spouse of patient P16).

### 5.4 Theme 4: Benefits Encouraged Maintenance in Behaviour Changes

All the participants reported at least one benefit from the hybrid PR programme. The majority of participants (85.7%) reported an improvement in their daily physical activity attributed to the intervention. Improvement in the 6‐min stepper test aligned with participants' perspectives (Table 2, *p* = 0.002).

The rehabilitation motivated me to start doing regular exercise again, which I probably wouldn't have done otherwise. (P3)

I can drive again, go shopping, sleep in the upstairs bedroom and get my tools from the basement. (P17)

Most of the participants reported that they had maintained their exercises training programme after the end of PR. The exercises were incorporated into their daily routines from the beginning of PR, facilitating maintenance in the absence of the care manager supervision.

I did the exercise sessions at home on my own, without my “coach”. At the end, I was able to carry on doing the exercises on my own. (P10)

In addition to physical benefits, 12 (57.1%) participants reported psychological improvements, including enhanced stress management and dyspnea, attributable to the self‐management strategies taught during the programme.

I'm very happy with the programme because it taught me stress management techniques that I use every day. (P10)

What I do every day now is cardiac coherence, breathing, staying calm. And honestly, belly breathing really does me a lot of good. (P16)

Moreover, 9 (42.8) participants reported improved self‐confidence and self‐esteem. Improvement in anxiety and depressive symptoms aligned with participant's experiences (Table 2, *p* = 0.001).

Before I started the programme, I felt worthless and had no confidence. After doing it, however, I found joy in life again. (P9)

Long‐term behavioural changes also include smoking cessation, increased regularity of GP visits, and improved vaccination records.

I see a doctor regularly now and have had my vaccine to reduce the risk of bronchitis. (P1)

Smoking is a distant memory. I don't smoke anymore. (P5)

However, the long‐term maintenance of healthy behaviours could be disrupted by the emergence of new comorbidities over time.

I stopped doing the exercises when I was diagnosed with cancer. (P8)

Furthermore, a majority of participants indicated that the number of PR sessions was not enough. Four patients reported that the limited number of sessions had a negative effect on their satisfaction or on benefits maintenance.

I didn't do the exercises regularly because the programme was too short. If nobody motivates you, you won't get anywhere with such a painful illness. (P14)

There should be more sessions and a follow‐up. Because it suddenly stops, and you go back to your old life. (P13)

These qualitative findings were supported by the satisfaction questionnaire, where the two items on programme duration and session frequency scored slightly lower, with a mean of 8/10.

### 5.5 Theme 5: Hybrid Programme Challenges

Five individuals experienced difficulties with the video call connection or during the calls. Furthermore, 17 (80.9%) participants expressed a strong preference for face‐to‐face visits, as they were regarded as more conducive to the supervision and monitoring of physical exercises, which were deemed to be safer with direct supervision (Table 6). They were also favoured by participants as they facilitated greater confidentiality and trust in the care manager. Despite their preference, participants acknowledged the utility of remote sessions for monitoring physical training adherence and changes in respiratory symptoms.

**TABLE 6:** Factors challenging the implementation of a hybrid PR programme.

**Technical challenges: telerehabilitation equipment and internet connection** I couldn't get online, so we did the session on the phone instead. (P12) Sometimes I had no signal because my village doesn't have good network coverage. (P1)
**Participants feel safer and more comfortable with face‐to‐face supervised visits** It was harder to do the exercises via the video call than when she was with me at home. (P7) It was reassuring to see the physiotherapist in person; the video calls alone wouldn't have been enough. (P3) This closeness made me feel comfortable enough to open up, but that's something you'd rather share in person. (P15)
**Perceived as ‘check‐up’ visits** The home supervised sessions were better, but the video calls were also useful, especially when I had specific questions about my symptoms or to report on my daily activities. (P10)

### 5.6 Quantitative Data Regarding the Effectiveness of the Hybrid Programme

Baseline and post‐intervention outcome values are presented in Table 2. Post‐intervention changes were both statistically and clinically significant for the CAT (mean Δ(SD): −5.7 (4.2), *p* < 0.001), HADS (mean Δ(SD): −6.0 (5.8), *p* < 0.001) and 6MST (mean Δ(SD): +92.0 (94), *p* = 0.002). Among the six patients who were unable to perform the stepper test at baseline due to physical frailty, five remained unable to complete it at the end of the programme.

## 6 Discussion

This study explored the experiences and satisfaction of people with COPD who participated in a hybrid home‐based PR programme following hospitalisation for an exacerbation. The home‐based option was well accepted and preferred by most participants over the centre option. Participants reported multiple benefits, including improvements in physical, psychological and quality of life outcomes. The factors mentioned as contributing to these benefits were as follows: (i) easy access and early personalisation of the programme to individuals' needs and goals; (ii) trust in a dedicated transdisciplinary care manager delivering the entire intervention; and (iii) the integration of the informal carer as an important source of emotional and physical support. Nevertheless, technical challenges were faced during the remotely supervised sessions, and participants consistently expressed a preference for face‐to‐face supervision over telerehabilitation sessions.

All participants were given the option by their physicians of performing the PR programme either at home or in a centre. As reported in the literature, the factors that influenced their decision included the absence of transportation, the possibility to keep‐up with professional and family life or the desire to limit other hospital visits (Cox et al. 2023; Lahham et al. 2018). PR prescriptions may be influenced by the physician's belief that inpatient care is more appropriate for this vulnerable population than home interventions; yet, as reported in the literature (Cox et al. 2022; Lahham et al. 2018; Wan et al. 2024), participants reported feeling safe during the home supervised training sessions and no adverse events were reported. Therefore, the experiences of participants suggest that this hybrid home‐based programme is safe and can enhance PR adherence in people with AECOPD.

The involvement of a single healthcare professional (a care manager), with specialised training in PR, throughout the entire intervention was key to facilitating the therapeutic alliance and trust. Although the care manager role is specific to our home‐based PR programme, a similar concept (i.e., the case manager) has been successfully implemented in hospital settings (Trappenburg et al. 2011) and may represent a preliminary step toward implementing a transdisciplinary care approach (Bourbeau et al. 2018; Campagna and Nelson 2019). However, in most interprofessional models described in the literature, case managers primarily coordinate care between disciplines rather than delivering integrated care across traditionally distinct professional roles. In contrast, our care manager model extends beyond coordination and may represent a further step toward a fully transdisciplinary care approach.

Most participants reported high satisfaction with the emotional and psychological support provided by the care manager. While anxiety and depressive symptoms are common in people with COPD (Yohannes and Alexopoulos 2014) and are associated with an increased risk of exacerbation, hospital readmission and mortality (Rahi et al. 2023), previous studies offering holistic care approaches have shown benefits (Ma et al. 2020; Yohannes et al. 2022). Our findings suggest that a hybrid, home‐based transdisciplinary programme can also lead to meaningful improvements in anxiety and depressive symptoms. The convergence of quantitative improvements and qualitative reports of emotional support underscores the added value of this care manager–led approach in a post‐hospitalisation context.

Nearly half of the informal carers (47.6%) were actively involved in the intervention, and participants reported high satisfaction with their involvement, particularly in terms of emotional support, motivation and practical assistance with remote sessions. This aligns with previous studies highlighting the key role of informal carers in promoting adherence to treatment and healthier behaviours in COPD (Mesquita et al. 2017; Trivedi et al. 2012), and in thus reducing the risk of exacerbations (Hobman et al. 2023). However, while informal carers play a crucial role during and after exacerbations, this involvement is also associated with increased burden, fatigue and psychological distress (Machado et al. 2022; Suresh et al. 2022). In line with earlier findings showing that PR may alleviate some of these negative effects on carers (Grosbois et al. 2022; Marques et al. 2021), carers in our study reported feeling emotionally supported by the care manager. Including carers in telerehabilitation sessions was more challenging than during face‐to‐face home visits, suggesting that remote formats may require specific adaptation to better accommodate carers.

Although the hybrid home‐based PR programme appears to be a promising alternative to centre‐based rehabilitation, it was not feasible for all participants in real‐life conditions. Barriers to telerehabilitation included access to appropriate devices, a reliable internet connection and digital literacy. In contrast, RCTs typically select participants who already meet these technical requirements (Bourne et al. 2017) or provide all necessary equipment (Cox et al. 2022; Neves et al. 2023). While such designs are essential to demonstrate efficacy, they may underestimate implementation barriers in routine practice. Our findings highlight the gap between controlled trial conditions and real‐world delivery, underscoring the importance of considering digital access and usability when scaling up hybrid PR programmes.

### 6.1 Strengths and Limitations

This mixed‐method study is the first to document the experience of people with COPD recently discharged after an exacerbation, regarding the hybrid home‐based PR programme that they completed. The mixed‐methods design was chosen to bridge the gap between observed outcomes and implementation processes, exploring both behavioural factors (e.g., participant engagement) and contextual factors (e.g., environmental or organisational influences) that facilitated or hindered success. Deductive thematic analysis was conducted by three authors, including one independent of the intervention (E.M.). While the participants were randomly selected from a list provided by the PR team, the main limitation of the study is that it only documents the experiences of those who have chosen to enrol and complete the hybrid programme. Data from the ongoing larger study indicate that 36% of eligible patients declined participation due to barriers such as visual or hearing disability, lack of internet access or fear of telerehabilitation sessions, highlighting the need to explore the perspectives of those who did not participate. In addition, the relatively small sample size may limit the robustness of the quantitative analyses; these findings should be confirmed in the larger ongoing prospective study. Although most patients began rehabilitation 6 weeks after hospital discharge, five started later due to delays in physician referrals, rather than the PR team, who typically initiated the programme within 2 weeks. Participants were not specifically asked about prior PR experience, which might have provided additional context for their preferences. The study was monocentric, conducted in northern France and interviews were performed in French; translation may have influenced some expressions, but primary themes and conclusions are likely unaffected.

## 7 Relevance to Clinical Practice and Policy

Telerehabilitation was developed to diversify care and increase access to PR, but the variety of tools remains a challenge for generalisation (Cox et al. 2021). In our study, participants strongly preferred face‐to‐face sessions, aligning with evidence that phone apps do not outperform standard care (Gloeckl et al. 2024). These findings, supported by other qualitative studies (Cox et al. 2023; Lahham et al. 2018), suggest that telerehabilitation should include at least some supervised individual or group face‐to‐face sessions. The balance between remote and in‐person sessions should be tailored to participants' needs and progress, within funding constraints. Although we did not conduct an economic evaluation, hybrid PR likely reduces costs compared with conventional centre‐based programmes while maintaining comparable short‐term outcomes. Overall, our results support investment in hybrid models, while further work is needed to refine their design and implementation.

## 8 Conclusion

This mixed‐methods study indicates that a hybrid home PR programme, combining face‐to‐face and remote supervised sessions, is mostly feasible, effective and well‐received by people with COPD who have been discharged from hospital following an exacerbation. Participants expressed their satisfaction with the flexibility of the hybrid home programme that was offered by a dedicated transdisciplinary care manager who demonstrated attentiveness to their and their carer's needs. However, significant limitations have been reported regarding the remote sessions, indicating that despite their effectiveness, telerehabilitation programmes are not accessible to all patients and should not be the only PR option offered.

## Author Contributions

S.G.: formal analysis and interpretation, writing the original draft. E.M.: acquisition of data, formal analysis and interpretation, writing the original draft. R.L. and O.L.R.: writing – review and editing. J.‐M.G.: conceptualization, interpretation, writing – review and editing. All authors gave final approval of the version to be published.

## Funding

The delivery of the home‐based PR programme was financially supported by the National Health Insurance Fund (Article 51, Innovative fund). The funder played no role in the design, conduct or reporting of this study.

## Ethics Statement

Human Research Ethics approval was provided by the observational research protocol evaluation committee of the French Language Society of Pulmonology (CEPRO 2021‐054) and utilisation of the data was authorised by the National Data Protection Commission (CNIL, Article 51).

## Conflicts of Interest

O.L.R. reports personal fees and non‐financial support unrelated to the submitted work from AstraZeneca, Boehringer Ingelheim, Chiesi, CSL Behring, GlaxoSmithKline, MSD France, Vertex and Vitalaire. O.L.R. is principal investigator in studies for Vertex and CSL Behring. J.‐M.G. reports personal fees and non‐financial support unrelated to the submitted work from AstraZeneca, Boehringer Ingelheim, Chiesi, CSL Behring, GlaxoSmithKlein, Menarini. The other authors declare no conflicts of interest.

## Data Availability

The data that support the findings of this study are available from the corresponding author upon reasonable request.
